# Male sexual orientation, gender nonconformity, and neural activity during mental rotations: an fMRI study

**DOI:** 10.1038/s41598-020-74886-0

**Published:** 2020-10-30

**Authors:** Monika Folkierska-Żukowska, Qazi Rahman, Artur Marchewka, Marek Wypych, Dawid Droździel, Andrzej Sokołowski, Wojciech Ł. Dragan

**Affiliations:** 1 Faculty of Psychology, University of Warsaw, Warsaw, Poland; 2 Department of Psychology, Institute of Psychiatry, Psychology and Neuroscience, King’s College London, London, UK; 3 Laboratory of Brain Imaging, Nencki Institute of Experimental Biology of the Polish Academy of Sciences, Warsaw, Poland; 4 Department of Neurology, Memory and Aging Center, UCSF Weill Institute for Neurosciences, University of California, San Francisco, San Francisco, CA USA

**Keywords:** Sexual dimorphism, Problem solving, Human behaviour

## Abstract

The cross-sex shift hypothesis predicts that gay men should perform more like heterosexual women on important neurocognitive tasks on which men score higher than women, such as mental rotation. Studies also suggest sex differences exist in the neural correlates of mental rotation. However, no studies have taken sexual orientation into account or considered within-group variation attributable to recalled gender nonconformity (a developmental trait reliably associated with human nonheterosexuality). We quantified the neural correlates of mental rotation by comparing two groups of gay men, gender conforming (*n* = 23) and gender nonconforming (*n* = 23), to gender conforming heterosexual men (*n* = 22) and women (*n* = 22). We observed a sex difference between heterosexual men and women in the premotor cortex/supplementary motor cortex and left medial superior frontal gyrus. We also observed a sex difference as well as a cross-sex shift in gay men who recalled being gender nonconforming as children in the right superior frontal gyrus, right angular gyrus, right amygdala/parahippocampal gyrus, and bilaterally in the middle temporal gyrus and precuneus. Thus, cross-sex shifts may be associated with underlying developmental factors which are associated with sexual orientation (such as gender nonconformity). The results also suggest that gay men should not be studied as a homogenous group.

## Introduction

Sex differences in certain cognitive functions are well documented. On average, women perform better than men on tests of verbal fluency, emotion recognition, and object location memory^1–5^. On average, men perform better than women on tests of spatial cognition, such as mental rotation and spatial perception (e.g., line orientation), and spatial memory tasks, such as navigation and route learning^4^. In particular, the sex differences reported on mental rotation tasks are robust, reliable, and large^4–6^ (*d*s between 0.56 and 0.73^5^).

Several neuroimaging studies have investigated sex differences (irrespective of sexual orientation) in neural correlates of mental rotation using Shepard and Metzler type tasks^7^. These studies reported differences between the neural activations found in men and women, even when there was no corresponding behavioural difference in task performance^8–14^. The most common findings were higher activations in parietal areas in men^8,9,13,14^ and higher activations in frontal areas in women^8,9,11^, which were suggested to correspond to women using more effortful top-down (or analytic/serial) processing and men using bottom-up (or gestalt) processing^9–11^. Nevertheless, two studies found higher activations in some parietal areas in women (with higher activations in other parietal areas in men)^11,12^ and two studies reported higher activations in males in the frontal areas^13,14^. These are only the most consistent findings—many of the studies have also found unreplicated differences in other areas. The results of the relevant papers are summarised in Table 1. In terms of volumetric studies, Koscik et al. reported that women had greater grey matter volume in the parietal lobe than men, while men had a proportionally greater surface area of the parietal lobe, which corresponded with poorer mental rotation performance in women and better performance in men, respectively^15^. Such morphological differences may even be present in childhood^16^.

**Table 1:** Summary of previous findings from fMRI studies about sex difference in brain activity during Shepard and Metzler type mental rotation task.

Paper	No of subjects	Mean age of subjects^a^	Controlled for IQ	Controlled for sexual orientation	Performance	Brain activity	
						Regions more active in men than women	Regions more active in women han men
Thomsen et al., 2000^10^/Hugdahl et al., 2006^8^^b^	6 men, 5 women	30 ± 10 years	No	No	No sex difference	R/L superior parietal lobule (BA 7)	R/L inferior frontal gyrus (BA 45)
Jordan et al., 2001^12^^c^	10 men, 14 women	Men: 25.8 ± 5.8 years, Women: 21.3 ± 3.2 years	No	No	No sex difference	L Precentral gyrus, L M1 Posterior intraparietal sulcus, R Parieto-occipital sulcus, R Precentral sulcus, M1	R Superior parietal lobule, R Posterior intraparietal sulcus, R Anterior intraparietal sulcus, L Inferior temporal gyrus, L Precentral gyrus, PMdc, L Anterior intraparietal sulcus, L Inferior parietal lobule, R Inferior temporal gyrus, R Middle occipital gyrus, R Precentral gyrus, PMdc
Weiss et al., 2003^11^	10 men, 10 women	University students	No	No	No sex difference	R/L inferior parietal gyrus	R/L superior parietal lobe, R inferior frontal gyrus
Levin et al., 2005^14^	7 men, 5 women	20.67 years	No	No	Men responded more accurately, No sex difference in reaction time	R Paracentral frontal lobe, L Parahippocampal gyrus, L Medial frontal gyrus, R Medial frontal gyrus, R Anterior cingulate gyrus, R Middle temporal gyrus, R Inferior parietal lobe, R Inferior frontal gyrus	L Parahippocampal gyrus
Butler et al., 2006^9^	12 men, 13 women	Men: 30.1, SD = 5.9, women: 28.6, SD = 7.5	No	No	No sex difference	R postcentral gyrus (BA 5), L ventral globus pallidus, L medial parietal/paracentral lobule (BA 5), L postcentral gyrus (BA 3), L precuneus (BA 7), Bilateral peri-midbrain	L dorsalmedial prefrontal cortex/superior frontal gyrus (BA 8), R dorsalmedial prefrontal cortex/superior frontal gyrus (BA 8), L inferior occipito-temporal cortex R temporal pole (BA 21)
Semrud-Clikeman et al., 2012^13^	20 men, 20 women	Graduate students	Yes^d^	No	No sex difference	L precuneus/posterior cingulate/cuneus R inferior frontal gyrus/middle frontal gyrus L middle occipital gyrus	–

^a^Different levels of detail provided based on the information given in each publication.

^b^These two publications seem to be based on the same fMRI study.

^c^This study actually included combined results for a Shepard & Metzler 3D mental rotation task, and 2D mental rotation tasks with letters and abstract shapes as stimuli.

^d^Wechsler Abbreviated Scale of Intelligence, no differences between groups.

Growing evidence supports the notion that people of different sexual orientations also differ in certain types of cognitive ability. Heterosexual men perform better, on average, than gay men on tests such as mental rotation, line orientation, and spatial navigation^17–25^. In general, the performance of gay men is similar to that of heterosexual women. One meta-analysis reported that gay men performed like heterosexual women on both male-favouring (e.g., spatial cognition) and female-favouring (e.g., verbal fluency) cognitive tests, while lesbian women tended to perform like heterosexual men only on male-favouring tests, with mean effect sizes of 0.46, 0.21, and 0.11, respectively^26^. Sexual-orientation-related differences are hypothesised to follow a “cross-sex shifted” or sex atypical pattern. The “cross-sex shift” hypothesis predicts that gay men and women will show patterns of cognitive function similar to their opposite-sex heterosexual counterparts^26^. Thus far, a large body of evidence about psychological, behavioural, and cognitive traits supports the cross-sex shift hypothesis^26,27^.

To date, no study has investigated sexual-orientation-related differences in neural activations during mental rotation tasks, however some post-mortem and neuroimaging studies have explored the relationship between sexual orientation and brain structure and function. Neuroimaging studies comparing heterosexual and gay men and women have reported various differences in structure and function, some of which could be considered to be cross-sex shifts. For example, the volumetric patterns of brain activity in both lesbian women and gay men^28^, or grey matter volumes in the perirhinal cortex in lesbian women^29^. Some of these effects seem to be unique to sexual orientation, such as cortical thickness in the orbitofrontal and visual areas^30^ as well as the anatomy of the corpus callosum in gay men^31^. Several small-scale neuroimaging studies have also suggested cross-sex shifts in homosexual men and women in brain responses to putative sex-specific pheromonal compounds^32,33^. However, these have not been replicated. Previous post-mortem studies also pointed to small sexual-orientation-related differences in hypothalamic regions and the anterior commissure; again, there are no robust replications of these^27^. All such studies are limited by small sample sizes, inconsistent reporting of methods for assessing sexual orientation, or failure to consider important confounders (such as general intelligence).

One important developmental factor associated with sexual orientation and, possibly, with sexual variation in cognition is childhood gender nonconformity (CGN). CGN refers to sex-atypical behaviours, interests, hobbies, activity levels, and play partner preferences before the age of 12. Both retrospective and prospective studies report a strong association between CGN and nonheterosexuality in adulthood^34,35^. These studies indicate that gay men are, on average, more feminine in behaviour and interests during childhood compared to heterosexual men, while lesbian women are more masculine, on average, in these respects compared to heterosexual women. It is possible that gender nonconforming behaviours during childhood may influence time spent on sex-typed activities (e.g., playing video games) which then influence cross-sex shifted cognitive differences in adulthood. Some research suggests that men may also typically spend more time on spatial activities, such as video games, which could be associated with better spatial abilities^36,37^. Several studies suggest that there are small associations between recalled CGN and performance in certain cognitive domains (e.g., object location memory^38^ and verbal intelligence^20^; cf. Rahman et al.^39^, and Rahman and Yusuf^40^). There are also indications that adult psychological gender traits are associated with mental rotation performance, with higher masculinity correlating with higher scores in both men and women^41^. These, albeit small, associations support the hypothesis that developmental CGN explains some of the variation in some behavioural correlates of sexual orientation (e.g., cognition)^42^. Factors such as genetic influences, prenatal sex hormones, or maternal immunization could comprise a common causal mechanism through which CGN is linked to sexual orientation and its neurocognitive correlates^27,42^.

Importantly, recent work suggests there is not one single developmental pathway to male sexual orientation, but rather several distinct ones^43^. Four biodevelopmental subgroups of male sexual orientation have been distinguished based on having certain developmental markers including fraternal birth order, handedness, and familial clustering of nonheterosexuality. Most heterosexual and nonheterosexual men were grouped in the profile that did not have any biomarker, and these also tended to be the most gender conforming. The three profiles linked to the biomarkers were mostly comprised of nonheterosexual men. This unique study suggests that there may be important and distinct subgroups within nonheterosexual men that require further study.

Given the current literature, the present study aimed to quantify the neural correlates of sexual-orientation-related differences in performance on mental rotation for the first time. Mental rotation tasks are the most appropriate because they show robust sex and sexual-orientation-related behavioural differences and are associated with a reliable pattern of neural activity. The present study will also look at sex differences in mental rotation in the biggest sample thus far. Previous studies used small samples (most had ≤ 10 individuals per group; only one study had 20 per group), and the areas in which differences were reported varied, making it hard to identify consistently reported regions of interest (ROIs) associated with these differences. It will also be the first study on neural correlates of sexual orientation to consider developmental factors associated with sexual orientation or possible subgroups within sexual orientation categories.

As part of this study (described in the Supplementary Material), we found in a sample of over 700 gay and heterosexual men that mental rotation performance was related to CGN rather than sexual orientation per se. Based on this, as well as previous indications that gender nonconformity may be an important correlate of individual differences in cognitive performance between and within the sexes, we hypothesised that gay men would score lower on mental rotations than heterosexual men and that individuals who report having been gender nonconforming in childhood would also score lower than those who report having been gender conforming. We tested these hypotheses in an offline behavioural study on a large sample of gay and heterosexual men (see the Supplementary Material). Next, we tested whether gay men show a cross-sex shifted pattern of neural activity while performing a mental rotation task in an fMRI scanner. We did this with two groups of gay men (gender conforming and gender nonconforming) and compared them with gender conforming heterosexual men and women. Specifically, we hypothesised that heterosexual men would show higher activations in the parietal region than heterosexual women and that heterosexual women would show higher activations in the frontal regions than heterosexual men. We further hypothesised that gender conforming gay men would show patterns of neural activations similar to those of heterosexual men and gender nonconforming men would show patterns of neural activations similar to those of heterosexual women. As such, our study considers both sexual orientation and gender (non)conformity and, thus, can help bring us closer to understanding which of these is more related to visuospatial skills. However, it cannot help settle the question of whether the observed differences are due to prenatal biological or postnatal psychosocial factors.

## Results

### Group characteristics

**Figure 1:**
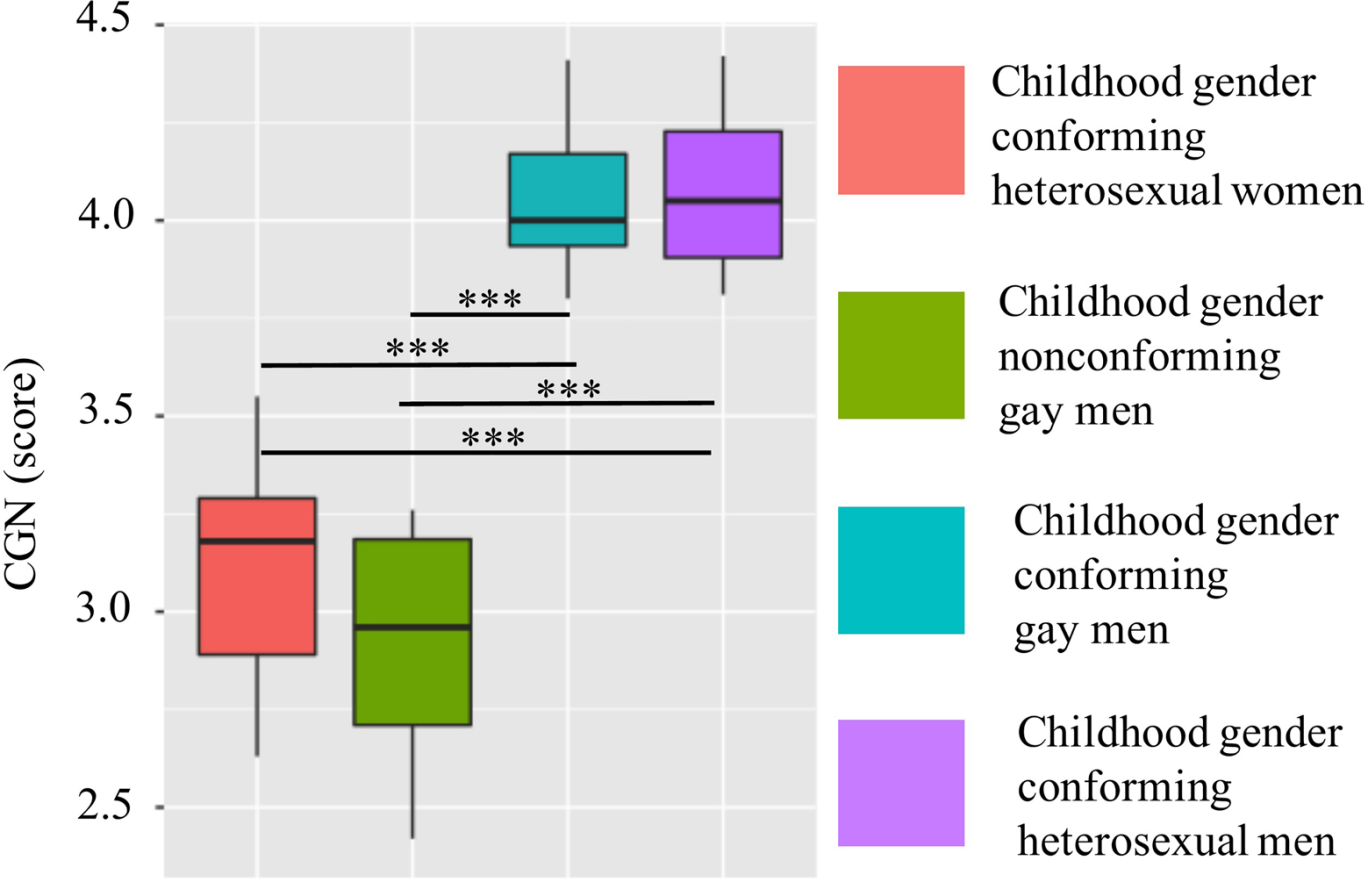
Boxplots representing CGN scores. Significant differences revealed with Tukey HSD pairwise comparisons are marked on the graph. *** significant at *p* < 0.001.

The groups differed significantly in their CGN scores as expected (*F*(3,85) = 163.2, *p* < 0.001). Tukey Honest Significant Difference (HSD) tests revealed no significant difference (*p* = 0.07) in terms of childhood levels of femininity/masculinity between childhood gender conforming heterosexual women and childhood gender nonconforming gay men and no significant difference (*p* = 0.898) between childhood gender conforming gay men and childhood gender conforming heterosexual men. These tests revealed significant differences in childhood femininity/masculinity levels between childhood gender conforming heterosexual women and both childhood gender conforming gay men and childhood gender conforming heterosexual men. Furthermore, a significant difference in childhood femininity/masculinity levels was found between childhood gender nonconforming gay men and both childhood gender conforming gay men and childhood gender conforming heterosexual men (all at *p* < 0.001; Fig. 1). The groups did not differ significantly in IQ scores (*F*(3,85) = 0.74, *p* = 0.71) or age (*F*(3,85) = 0.45, *p* = 0.72). See Table s3 in the Supplementary Material for mean scores and standard deviations of these measures for each group.

### Behavioural results (in-scanner performance)

Mean scores and reaction times for these can be found in Table s2 of the Supplementary Material.

**Figure 2:**
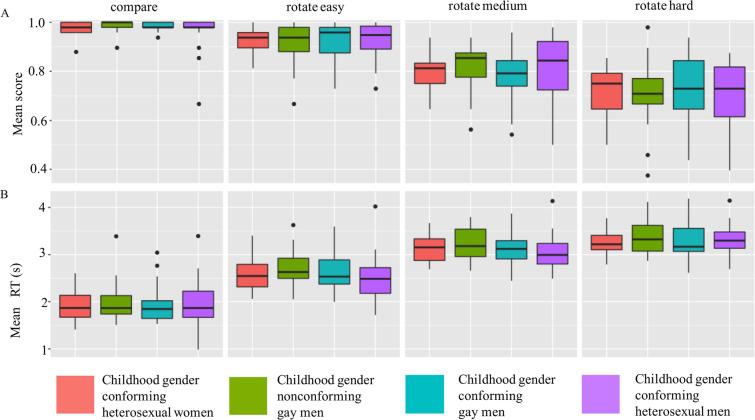
Boxplots representing scores (**A**) and reaction times (**B**) in the four groups for each task difficulty level.

A two-way mixed ANOVA revealed a significant effect of difficulty level (*F*(3,255) = 150.16, *p* < 0.001) but no significant group effect (*F*(3,85) = 0.131, *p* = 0.941) or significant group–condition interaction (*F*(9,255) = 0.454, *p* = 0.866) for mental rotation scores (Fig. 2A). Greenhouse–Geisser correction was applied to all results, as Mauchly’s test revealed that the data were non-spherical.

Similarly in the case of mental rotation reaction times, there was a significant effect of difficulty level (*F*(3,255) = 877.14, *p* < 0.001), but no significant group effect (*F*(3,85) = 0.457, *p* = 0.713) or significant group–condition interaction (*F*(9,255) = 1.36, *p* = 0.206; Fig. 2B). Greenhouse–Geisser correction was applied to all results, as Mauchly’s test revealed that the data were non-spherical.

### Brain activations: main effect of task

**Figure 3:**
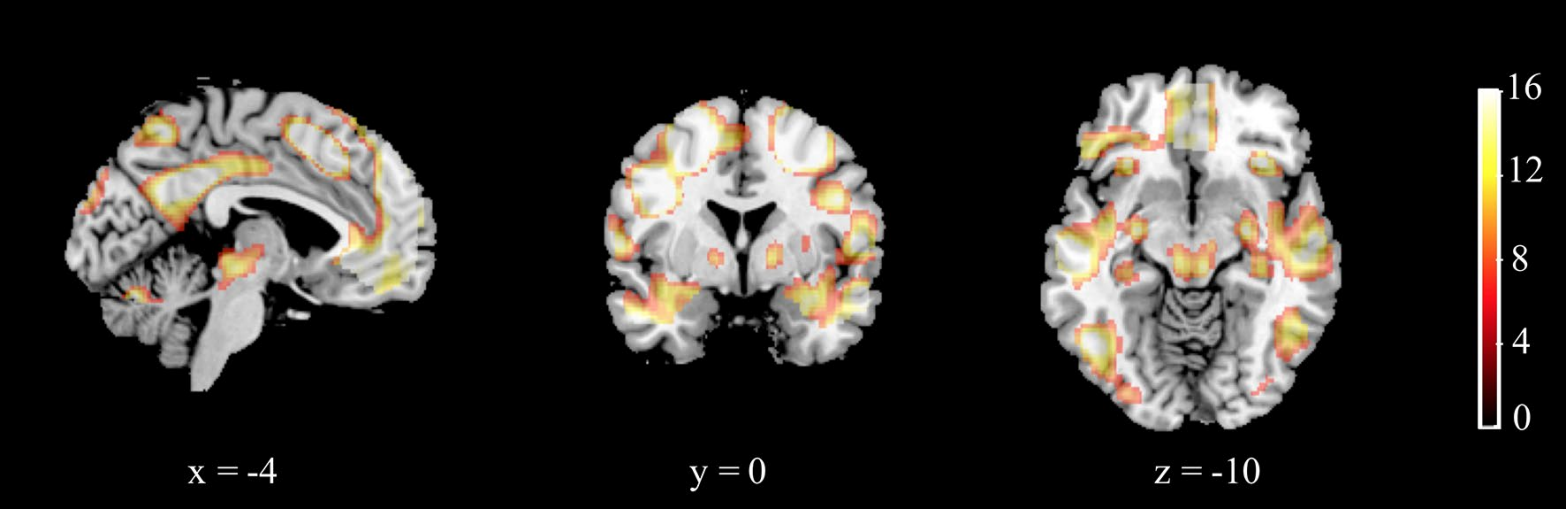
Main effect of task. The colour bar represents *F* values; x, y, and z represent Montreal Neurological Institute (MNI) space coordinates for the given slice. A voxel-wise height threshold of *p* < 0.001 (uncorrected) combined with a cluster-level extent threshold of *p* < 0.05 (corrected for multiple comparisons using the FWE rate) was applied.

A whole-brain *F*-test on the entire sample with 4 conditions (compare, rotate easy, rotate medium, and rotate hard) revealed the activations in the brain network presented in Fig. 3 and Table 2.

**Table 2:** Task effects: results of whole-brain one-way ANOVA.

Brain structure	Cluster Size (voxels)	*F* (peak)	MNI coordinates of the peak (mm)		
			*x*	*y*	*z*
**F-test—main effect of task**					
R superior frontal gyrus	17,884	73.81	26	− 2	54
L superior parietal lobule	13,721	69.73	− 38	− 44	48
R anterior insula	912	60.03	30	26	0
L anterior insula	796	57.28	− 30	24	0
L angular gyrus	7017	32.07	− 52	− 62	36
L/R medial superior frontal gyrus	8492	31.76	− 8	50	26
L/R posterior/middle cingulate gyrus	2617	23.32	− 12	− 48	34
R middle frontal gyrus	882	15.84	36	38	28
L/R cerebellar vermal lobules VI–VII	286	14.95	− 6	− 72	− 24
L/R Thalamus	1808	14.78	12	− 20	12
R inferior temporal gyrus	364	12.1	50	− 56	− 10
L/R Cuneus	264	9.98	− 2	− 94	24
**Post-hoc (t-test) rotate > compare**		t (peak)			
L/R superior frontal gyrus	15,892	13.28	26	− 2	54
L/R superior parietal lobule	18,192	13.26	− 38	− 42	46
L/R Thalamus	2486	6.02	12	− 20	12
**Post-hoc (t-test) compare** **>** **rotate**					
R angular gyrus	7737	8.87	58	− 62	26
L Superior Frontal Gyrus	8783	8.69	− 16	34	56
L angular gyrus	6812	8.53	− 52	− 62	36
L/R posterior/middle cingulate gyrus	3284	7.12	− 4	− 44	34

**Figure 4:**
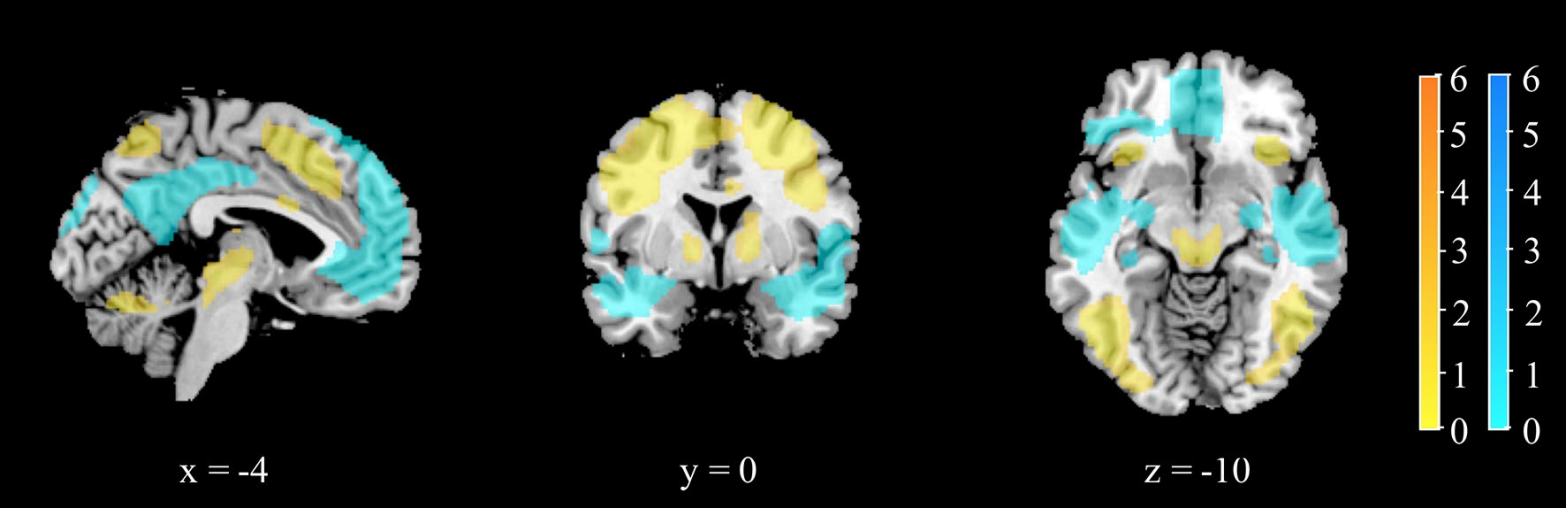
Results of the post-hoc *t*-test analyses for the *rotate* > *compare* contrasts and the *compare* > *rotate* contrast. The colour bar represents *t* values; clusters more active in the rotate conditions than in the compare condition are indicated in shades of yellow; clusters more active in the compare condition than in the rotate conditions are indicated in shades of blue; x, y, and z represent MNI space coordinates for the given slice. A voxel-wise height threshold of *p* < 0.001 (uncorrected) combined with a cluster-level extent threshold of *p* < 0.05 (corrected for multiple comparisons using the FWE rate) was applied.

Post-hoc analysis using a *t*-test identified the structures which were more activated in the rotate condition versus the compare condition as well as those which were less activated (Fig. 4 and Table 2).

### Brain activations: group differences

**Figure 5:**
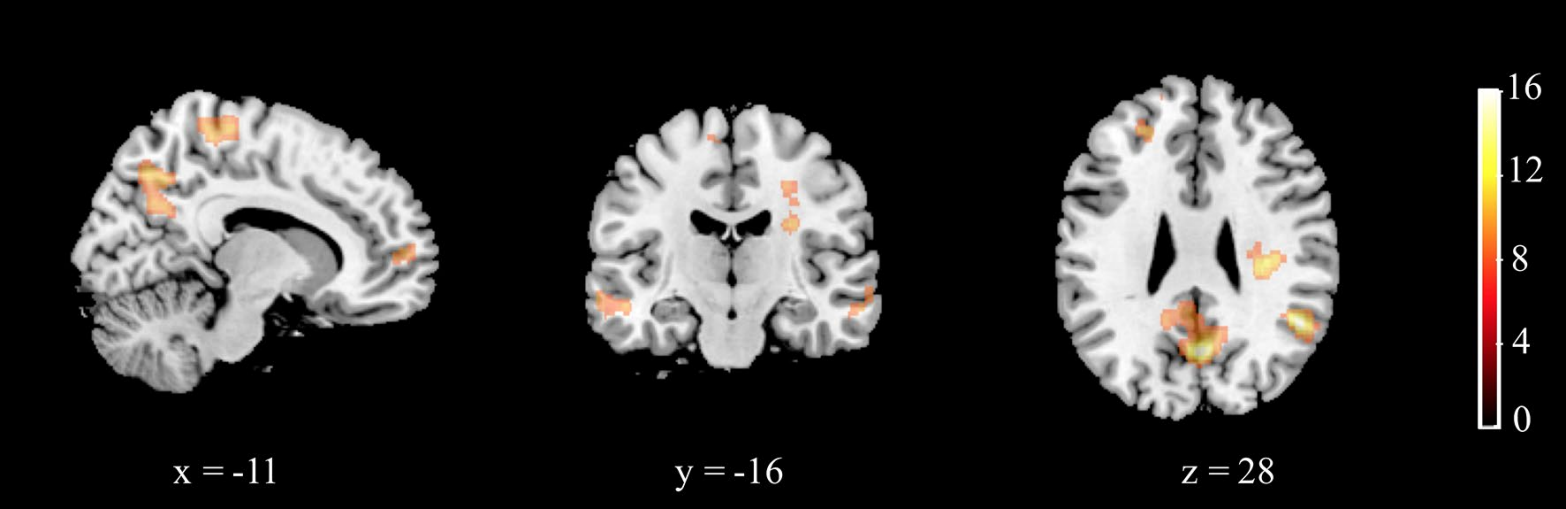
Main effect of group in the *rotate* > *baseline* contrast. The colour bar represents *F* values; x, y, and z represent MNI space coordinates for the given slice. A voxel-wise height threshold of *p* < 0.001 (uncorrected) combined with a cluster-level extent threshold of *p* < 0.05 (corrected for multiple comparisons using the FWE rate) was applied.

Following previous mental rotation literature (e.g., Butler et al., 2006), rotate conditions were combined for group comparisons. Whole-brain analysis (*F*-test, height threshold: *F*(3,86) = 5.93, *p* < 0.001, with FWEc correction) revealed a significant main effect of group in *rotate (easy* + *medium* + *hard)* > *baseline* in several structures (Fig. 5, Table 3).

**Table 3:** Group effects: results of whole-brain one-way ANOVA in the rotate > baseline contrast.

Brain structure	Cluster size (voxels)	*F* (peak)	MNI coordinates of the peak (mm)		
			*x*	*y*	*z*
L/R precuneus	1879	13.08	4	− 66	30
R angular gyrus	233	12.75	50	− 52	28
R middle temporal gyrus	217	10.76	58	0	− 24
L/R precentral/paracentral gyrus	495	10.28	-8	− 30	66
L middle temporal gyrus	318	9.77	− 52	− 12	− 14
R amygdala/parahippocampal gyrus	179	9.56	26	6	− 18
L medial superior frontal gyrus	188	9.33	− 16	52	20
R superior frontal gyrus	197	8.6	12	56	6

**Figure 6:**
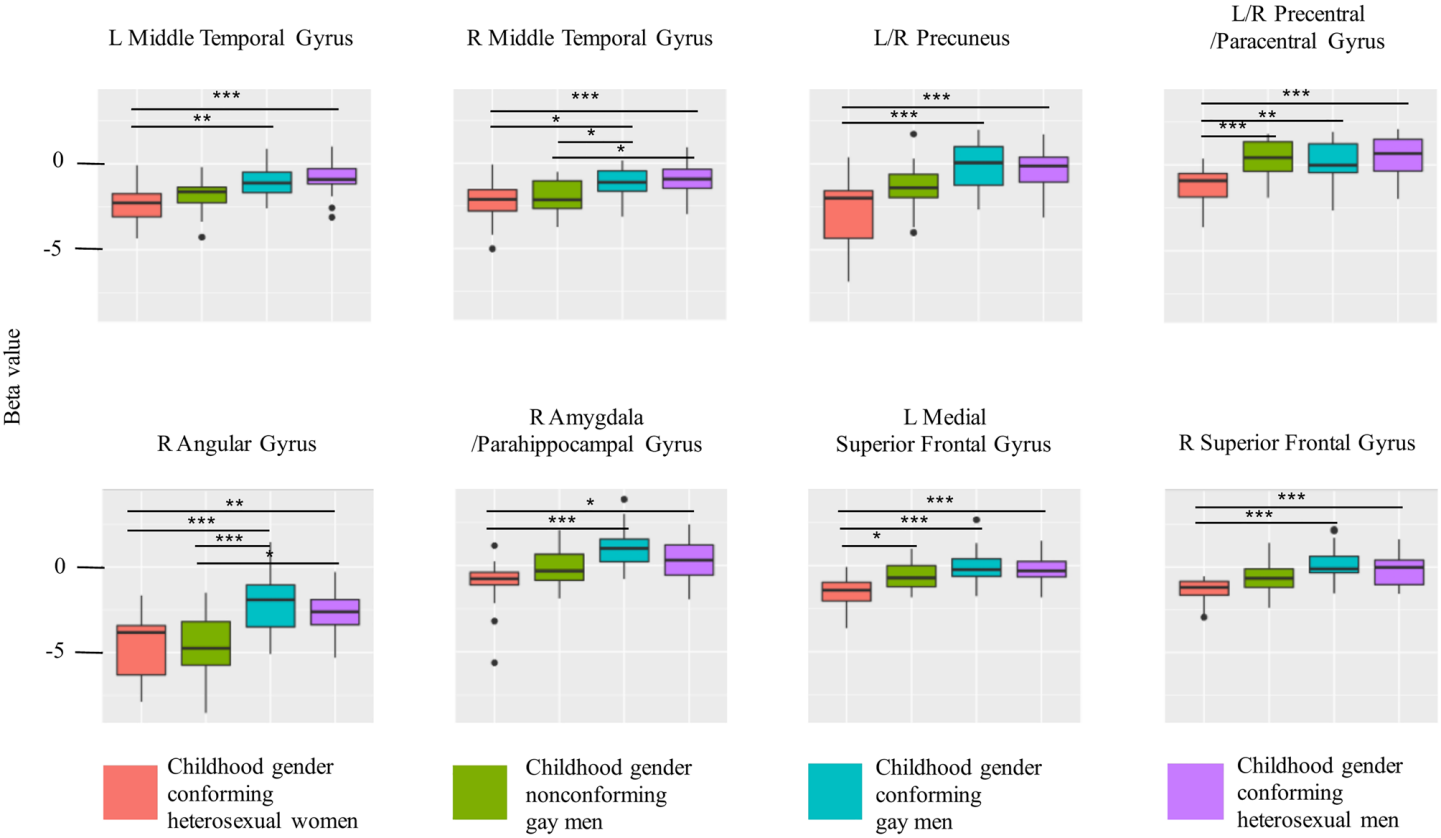
Boxplots representing beta values for mean activations for all levels of difficulty (*rotate* > *baseline* contrast) in the four groups in the regions in which a main effect of group was detected by the *F*-test. Significant pairwise Tukey HSD comparisons are marked on the graph. Results were corrected for multiple comparisons using Bonferroni correction. ***significant at *p* < 0.001, **significant at *p* < 0.01, *significant at *p* < 0.05.

Region-of-interest analysis of the clusters in which a main effect of group was observed revealed significant group differences. Results are presented in Fig. 6 and Table s3 in the Supplementary Material.

## Discussion

This study investigated whether the previously reported cross-sex shift in performance on the mental rotation task in gay men could be observed in terms of neural activations. In a behavioural study (described in the Supplementary Material) we found that CGN, but not sexual orientation, explains some of the variance in mental rotation performance. This result indicates that the cross-sex shift observed in previous studies may not be related to sexual orientation per se, but rather to underlying developmental factors which contribute to sexual orientation (namely, childhood gender nonconformity).

This served as a basis for the design of the current study, where, using fMRI, we compared neural activations during a mental rotation task between gender nonconforming gay men, gender conforming gay men, and gender conforming heterosexual men and women. Based on previous results regarding sex differences, we hypothesised that heterosexual men would show stronger activations in the parietal lobe than heterosexual women and heterosexual women would show stronger activations in the frontal lobe than heterosexual men. We also hypothesised that this pattern of differences would be cross-sex shifted in gay men who recalled having been gender nonconforming in childhood, but not in those who reported having been gender conforming. Our hypotheses regarding parietal regions were largely confirmed; however contrary to our expectations, we observed stronger frontal activations in men than in women (with only a partial cross-sex shift in gender nonconforming gay men). Nevertheless, we also observed significant differences in activations between the groups in other regions, some consistent with our hypothesis regarding the cross-sex shift, which is discussed in more detail below.

Performance on the mental rotation task in the scanner decreased with increasing difficulty, which indicates that the difficulty levels were valid. Whole-brain analysis of the task effect, performed irrespective of sex and sexual orientation, revealed a network of task-related activations that were largely consistent with previous findings^44^.

There were strong positive activations associated with mental rotation bilaterally in the parietal lobe, superior frontal gyrus, and thalamus. The activations in the right angular gyrus, left superior frontal gyrus, left angular gyrus, and bilaterally in the posterior/middle cingulate gyrus were higher in the compare condition, when there was no need for mental rotation. The network of structures in which activations decreased during mental rotation corresponds partially to the default mode network, which could indicate increasing attentional focus with increasing task difficulty or less time spent on mind-wandering, as reaction times increased with task difficulty^45^.

There were no group differences in mental rotation performance in the scanner. However, this might be due to the sample size being too small to detect such behavioural differences. Nevertheless, this allowed us to focus on group differences in neural activations without them being confounded by performance differences or group differences in learning (and groups were matched in terms of general intelligence). In terms of the hypothesised group differences in neural activations, we focused on rotate versus baseline contrasts in a whole-brain analysis, consistent with previous approaches^9,46,47^. Tests for main effects showed group differences in clusters bilaterally in the precuneus, right angular gyrus, right middle temporal gyrus, bilaterally in the medial segment of the precentral gyrus, left middle temporal gyrus, in a cluster extending from the right amygdala to right parahippocampal gyrus, the left medial superior frontal gyrus, and right superior frontal gyrus. The structures in which we observed group differences only partially overlap with the structures where sex differences have been previously reported^8–14^.

Post-hoc region-of-interest group comparisons revealed that both of the gender conforming groups of men had higher activations than gender conforming women in the precuneus (part of the superior parietal lobule), resembling some of the previous reports of sex differences^8,9,13^. This is somewhat consistent with our hypothesis about the role of childhood gender nonconformity in any cross-sex shift. The superior parietal lobule is the structure most consistently reported as central to mental rotation^44^. In general, many fMRI studies on sex differences in neural correlates of mental rotation report higher activations in the parietal lobe in men, but no such study has explicitly measured the sexual orientation of the male and female participants^8,9,13^. A similar pattern of group differences was observed for the right amygdala/parahippocampal gyrus. Previous studies have shown that the activity of the amygdala can modulate the activity of the superior parietal lobe through connection with subcortical structures, facilitating more efficient mental rotation^48^, which could explain the similar pattern of group differences in the two areas.

Childhood gender nonconformity was also associated with both the left and right middle temporal gyrus activations, where we additionally observed a statistically significant difference between heterosexual men and gender nonconforming gay men. Higher activation in this region during mental rotation in men than in women has also been previously reported^14^. This pattern, together with the pattern observed in the right angular gyrus (a region important for number processing and spatial cognition^49^) is the most consistent with our hypotheses regarding the cross-sex shift. Here too, in addition to the fact that only gender conforming men differed from women, there was also a significant difference between the two groups of gay men, and heterosexual men differed from gender nonconforming gay men. Sex differences in the anatomy and function of the right angular gyrus have been previously reported: one study found higher activations in this region in men than women during performance of mathematical tasks, but greater volume and grey matter density in this area in women^50^. However, our result is inconsistent with that of Butler et al.^9^, who demonstrated higher activations in the angular gyrus in women than in men during mental rotation; furthermore, this result is surprising in the context of the task-effect analysis, which indicated that it was more active when there was no rotation.

In the right superior frontal gyrus, only the gender conforming men differed from women, and their activations were higher, which is inconsistent with some previous studies^8,9,11^, which found higher activations in frontal areas in women than in men. This was interpreted as indicating the use of a top-down processing strategy (in contrast to the bottom-up strategy employed by men). However, our results are in-line with studies which showed higher activations bilaterally in the frontal gyrus in men than in women during mental rotation^12–14^. However, the differences in the left medial superior frontal gyrus were limited to between-sexes, with lower activations in women than in all groups of men. Similarly, in the area of the precentral/paracentral gyrus corresponding to the premotor cortex and supplementary motor cortex (areas that are also crucial to mental rotation^51^), only a sex difference was observed, with all groups of men characterised by similar activations—all higher than those observed in women.

Since we did not measure spatial strategies in our study, we cannot infer whether strategy differences explain some of the group differences observed. However, given that we conducted the fMRI scans under conditions of no behavioural differences in mental rotation performance, and yet we observed differences in neural activations, it is possible that the groups may have employed different strategies to solve the task. Previous studies suggest that the different strategies used to solve mental rotation tasks may be associated with different patterns of brain activations^44^.

The patterns of group differences suggest that the cross-sex shift is substantially associated with gender nonconformity, rather than sexual orientation per se—higher activations in the identified structures largely correspond to higher masculinity. The results of our study fit well with the idea of the existence of sub-groupings within sexual orientation categories^43^.

Theoretically speaking, there are several hypothesised developmental mechanisms thought to be involved in sex and sexual-orientation-related neurocognitive differences. These primarily include hormonal and psychosocial influences. Prenatal androgens may act on the developing brain to organise these differences^52^. Other suggested mechanisms involve learning or gender-related socialization. On one hand, it has been found that mental rotation performance in boys as young as 5 months old correlates positively with perinatal testosterone levels^53^. On the other hand, parents and peers may encourage more spatial play in boys than in girls^54–56^. Interestingly, one study suggested that mental rotation performance in 5-month-old girls (but not boys) was correlated with the stereotypicality of parents’ attitudes about gender. Some studies on infants show that boys have an advantage on the mental rotation task as early as between 3 and 13 months, reporting medium effect sizes (η^2^p between 0.07–0.015^57–59^), which could be explained by both biological and social influences (however, some studies did not find this^60^). At present, it is not clear how socialisation-related factors would operate with respect to known sexual-orientation-related differences. One might expect that heterosexual and gay men are gender socialised in the same manner, yet they still show differences in certain areas of cognitive ability (heterosexual men being more male-typical and gay men being more female-typical). Based on our results, we cannot know whether cross-sex shifts in brain activations were more likely in gender nonconforming gay men than in gender conforming gay men due to prenatal or postnatal (including socialisation) factors. However, there are previous indications that childhood gender role behaviours are associated with prenatal factors (such as androgen exposure^61^ or maternal immunity^43^), and our results warrant further investigation of this matter. As well as a direct effect of biological influences on both childhood gender nonconformity and mental rotation abilities, there could be an indirect effect of biological influences on childhood gender nonconformity, which, in turn, would lead to differences in psychosocial factors that affect mental rotation (e.g., being interested in video games or other activities that facilitate the development of spatial abilities).

The present study has several important strengths. Our samples were well characterised in terms of sexual orientation (using multiple dimensions on a standardised measure) and levels of gender nonconformity (using a standardised measure). This has never been done before. Previous studies on sex differences simply assumed (or did not mention) that their male and female participants were heterosexual. Our participants were matched in terms of important confounders, such as age and IQ scores. Moreover, in order to increase statistical power, we defined our CGN groups based on k-cluster analysis, dividing a sample of almost 600 men into three clusters and recruiting from the two extreme clusters. We developed and used a robust and validated mental rotation task. The lack of mental rotation task differences in the fMRI part of this study allowed us to investigate putative group differences, unaffected by task performance or task-related learning processes. This allowed us to better quantify possible differences in basic or fundamental neural processes involved in task completion.

Nevertheless, there are several noteworthy limitations. Our study did not include lesbian women. Furthermore, despite the fact that we conducted a k-cluster analysis to create two subgroups of gay men on each extreme of the CGN spectrum, we had to include several participants from the low-scoring end of the middle group. We did not measure the strategies used by the participants when performing the mental rotation task, and thus we cannot know if the observed differences in activations are due to differences in strategies. Participants were not explicitly instructed when to ‘rotate’ and when to ‘compare’ the figures (cf. Butler et al.^9^). In our study, participants might also have attempted to rotate the figures they were meant to compare, thus we couldn’t rely on the rotate versus compare contrast in our analyses and had to also contrast rotation with baseline. While this approach has been used in many previous studies^9,46,47^, it is possible that we observed results that are not fully specific to the process of rotation, but are also common to other aspects of the visual processing of the stimuli. However, it is important to note that this limitation is consistent across groups. The fact that participants practiced the mental rotation task a day before the fMRI scan could be related to the lack of behavioural difference found here. There are also some indications that performance on the mental rotation task may be influenced by circulating testosterone levels as well as menstrual cycle phase^62^, neither of which were controlled in our study. Finally, as the participants were not informed about the hypotheses before the study and were generally naive about the study goals, it is unlikely that they could have been primed in a way that would trigger implicit gender stereotypes about performance on visuospatial tasks. Nevertheless, we cannot exclude the possibility that some spatial anxiety may have played a role in the performance of female participants in particular.

## Conclusion and future directions

We found evidence that a cross-sex shift occurs in gender nonconforming gay men only in several theoretically important brain regions of interest. Namely, in the right superior frontal gyrus, bilaterally in the middle temporal gyrus and precuneus, as well as the right angular gyrus and right amygdala/parahippocampal gyrus. This indicates that further investigations of differences *within* sexual orientation categories are warranted. Indeed, there is growing evidence that gay men are not a homogenous single category but may be characterised by distinct biodevelopmental sub-groupings^43^. Future studies should also aim to better quantify sex and sexual-orientation-related differences in brain activations when performing mental rotation using fully factorial designs (including lesbian women) and test the relationship between behavioural, strategy, and neural activity patterns when performing mental rotation in well-powered samples.

## Methods

### Participants

We decided to study men because effect sizes for gay men performing similarly to heterosexual women on spatial tasks are much greater than those for lesbians outperforming straight women (e.g., see meta-analyses by Xu et al., 2017, 2020^26,63^). We divided them into subgroups based on recalled childhood gender nonconformity and included only heterosexual women as a comparison group; lesbian women were not studied.

Gender nonconforming gay men (*n* = 23, mean age: 27.09, *SD* = 3.95), gender conforming gay men (*n* = 23, mean age: 26.26, *SD* = 4.48), gender conforming heterosexual men (*n* = 22, mean age: 25.59, *SD* = 3.65), and gender conforming heterosexual women (*n* = 22, mean age: 26.33, *SD* = 3.48) took part in this study. All study participants were of Polish nationality and were ethnically white. Behavioural data (scores and reaction times) from one subject from the heterosexual male group were excluded from analyses due to technical issues with the answering pad during the scan.

### Recruitment procedures

Male participants were recruited in Poland via an online survey completed as a part of a larger project concerning correlates of sexual orientation. The survey was distributed through Facebook (paid adverts and organic traffic), leaflets, posters, and word of mouth. Male participants for the fMRI study were recruited from the sample of men who had completed the behavioural study (described in Supplementary Material) and had attended the laboratory meeting before June 2018 (*N* = 594; of whom, 369 were gay and 226 heterosexual), and consented to be contacted about the fMRI study. For this study, participants were divided into three clusters using k-means cluster analysis based on their scores on the Recalled Childhood Gender Identity/Gender Role Questionnaire. There were 70 (18.97%) gay participants and one (< 0.01%) heterosexual participant in the highly gender nonconforming cluster, 191 (51.76%) gay and 50 (22.12%) heterosexual men in the middle cluster, and 108 (29.27%) gay and 174 (76.99%) heterosexual men in the highly gender conforming cluster. Gay participants were recruited from the highly conforming and nonconforming clusters, while heterosexual participants were recruited only from the highly gender conforming cluster. They were asked whether they would be willing to participate in an fMRI study and were interviewed regarding fMRI exclusion criteria. Because it proved impossible to recruit all 23 gender nonconforming participants from the appropriate cluster, 10 participants were recruited from among those with the lowest CGN scores in the middle cluster. Groups were matched as they were recruited such that there were no statistically significant differences in terms of age or IQ scores. Gender conforming female participants were recruited via a separate on-line questionnaire, advertised on-line (in the same way as the study pertaining to men), which included the same measures of sexual orientation, gender, and CGN, as well as demographic questions and questions regarding MRI exclusion criteria. Based on their age, psychological gender, and levels of CGN, they were invited to take the pen-and-paper IQ test. Women were then invited to the fMRI study such that the female group matched the male groups. All participants who completed the fMRI scan received a remuneration of 135 PLN (about 30 euros). Information about the remuneration was mentioned in the advertising materials.

### Behavioural measures

Sexual orientation was assessed using a Polish adaptation^64^ of the Sell Assessment of Sexual Orientation^65^. This tool measures three dimensions of sexual orientation: sexual attractions, sexual contact, and sexual identity. Homosexuality and heterosexuality are measured separately. For the purposes of this study, we defined predominantly heterosexual men as those who had been sexually attracted to either 0 or 1 men in the past year; who had never been attracted to a man or were attracted to a man less than once per month; whose attraction to a man in the past year ranged from “not at all” to “mildly”; who had been sexually attracted to at least one woman in the past year; who had been sexually attracted to a woman at least once a month; and who identified as “not at all homosexual” or “slightly homosexual” and as either “very heterosexual” or “extremely heterosexual”. Criteria for predominantly homosexual men were, mutatis mutandis*,* identical. Questions about sexual contact were not included in our criteria, as they have the limitation of being potentially constrained by social censure of same-sex sexuality. Only cis-gender men (i.e., men who reported being assigned male at birth and currently identified as male) took part.

Recalled childhood gender nonconformity was measured using a Polish adaptation of the Recalled Childhood Gender Identity/Gender Role Questionnaire^66^. The questionnaire was adapted for this study with the involvement of its author, and was translated and then back-translated. Only items corresponding to Factor 1 of the scale (concerning gender conformity) were analysed. An example of an item (and its associated point score) would be “As a child, the characters on TV or in the movies that I imitated or admired were: a. always girls or women (1), b. usually girls or women (2), c. girls/women and boys/men equally (3), d. usually boys or men (4), e. always boys or men (5), f. I did not imitate or admire characters on TV or in the movies”. A mean score was calculated for each participant for which the absolute range was 1–5, with 1 corresponding to extremely feminine behaviour and 5 to extremely masculine behaviour (in the original, scoring for women is reversed; we inverted it, so that scoring was identical for both sexes). A response of “I did not do this/feel this way” to an item was omitted. Since the questioning of traditional gender roles has intensified in recent years, for clarity we modified the instructions on the questionnaire by adding ‘In the case of the words "feminine" and "masculine", we refer to their stereotypical definitions functioning in society.’ The Cronbach’s alpha in our sample was 0.91.

General intelligence levels (IQ) were measured using the first half of the pen-and-paper version of the Polish adaptation^67^ of Cattell’s Revised Culture Fair Intelligence Test (CFT20R), with a maximum possible score of 56.

### Mental rotation task

**Figure 7:**
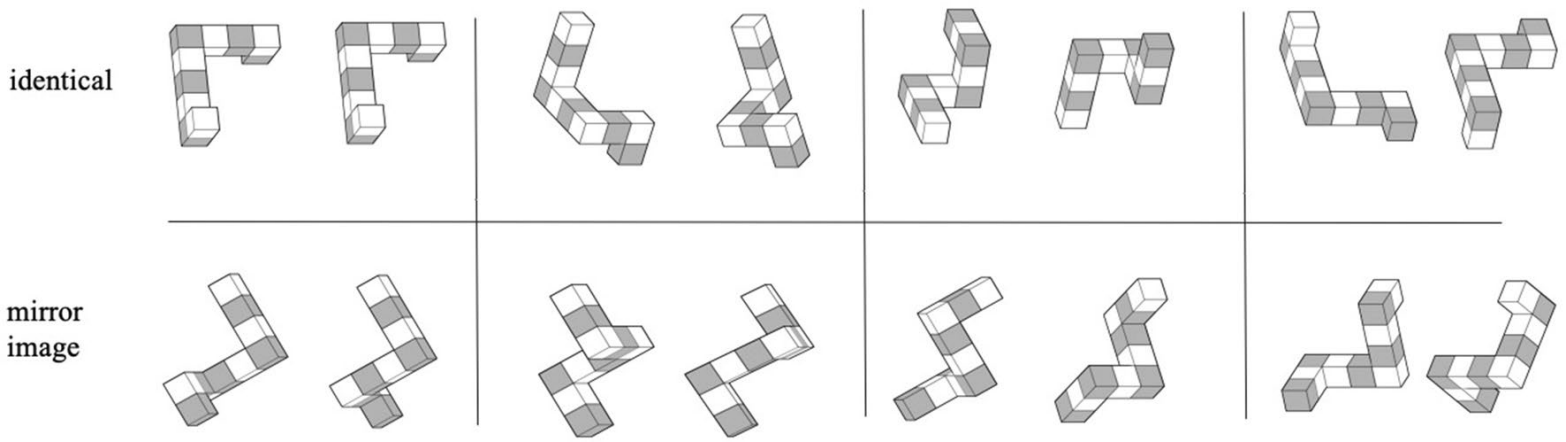
Sample stimuli from the mental rotation task solved by participants during the fMRI scan.

The experimental procedure was prepared using Presentation software (Version 20.1, Neurobehavioral Systems, Inc., Berkeley, CA, www.neurobs.com), and was based on the Shepard and Metzler task and its previous adaptations for fMRI experiment procedures^7,9^. Participants assessed whether two 3D figures^68^ presented next to one another on a grey background were identical or mirror images at four difficulty levels: compare, rotate easy, rotate medium, and rotate hard (rotations at 0°, 20°–60°, 80°–120°, and 140°–180°, respectively). Stimuli were presented in a block design with 24 pseudo-randomised blocks. Each block consisted of eight 5-s trials of the same difficulty level. Each trial consisted of 1 s fixation cross, 5 s stimulus presentation, and an inter-trial interval of a randomised duration between 1.5 s–3.5 s. Blocks were separated by 10 s breaks. The entire procedure was delivered over 4 fMRI sessions of six blocks each (counterbalanced in terms of the number of tasks of each difficulty per session) and lasted a total of about 30 min. Participants had to provide their answers during the 5 s of stimulus presentation and could score either 0 (incorrect or timeout) or 1 (correct) on each trial. The procedure was preceded by training, which consisted of 10 trials at various difficulty levels and with feedback (correct/incorrect/timeout). Participants completed the training twice (once outside the scanner and once inside the scanner) in order to familiarise themselves with using the response pads. No feedback was given during the actual test. The paradigm is available upon request from the corresponding author. Sample stimuli are presented in Fig. 7.

Note that in order to minimise potential performance bias due to men having previously performed the mental rotation test in the behavioural study described in the Supplementary Materials, all participants solved the Vanderberg and Kuse mental rotation test^69^ as practice one day before the scan.

### fMRI data acquisition

The participants performed the mental rotation task during a functional scan in a 3 T Siemens MAGNETOM Trio system (Siemens Medical Solutions) equipped with a 12-channel head coil. The functional scan (EPI images—TR: 2500 ms, TE: 28 ms, flip angle: 80°, voxel size: 3 × 3 × 3 mm, field of view: 216 mm, measurements: 240) was accompanied by a structural scan (T1-weighted image—TR: 2530 ms, TE: 3.32 ms, flip angle: 7°, voxel size: 1 × 1 × 1 mm, field of view: 256 mm) and a field map scan (TR: 800 ms, TE: 6.81 ms, flip angle: 60°, voxel size: 3 × 3 × 3 mm, field of view: 216 mm).

### Behavioural analyses (in-scanner performance)

The behavioral analysis was included to assess whether the sex and sexual-orientation-related differences in task performance were replicated in the present study. Behavioural differences between groups in terms of scores and reaction times for the mental rotation task performed during the scans were analysed using a two-way mixed ANOVA in IBM SPSS.

### fMRI preprocessing and analyses

The DICOM series were converted to NIfTI using the *Horos (Osirix) Bids Output Extension* (https://github.com/mslw/horos-bids-output), which is based on the dcm2niix converter (https://github.com/rordenlab/dcm2niix). The fMRI images were preprocessed and analysed with SPM12 (https://www.fil.ion.ucl.ac.uk/spm/). The preprocessing pipeline included: correction of inhomogeneous static magnetic field-induced distortion; correction for head movement—realignment to the first image; coregistration of the anatomical image to the mean functional image; segmentation of the coregistered structural image with the default tissue probability maps; normalisation to the Montreal Neurological Institute (MNI) space (voxel size after normalisation: 2 × 2 × 2); and smoothing with an 8 mm FWHM Gaussian Kernel. The Artifact Detection Toolbox (ART; https://www.nitrc.org/projects/artifact_detect/) was used to identify motion outliers (translation threshold: 2 mm; rotation threshold: 0.02 radians). The resulting regressors were added into the first level models.

General linear modeling was used to model the task-related differences. The model included onsets and durations of blocks of each difficulty level (compare and all rotate conditions) and the presentation of a fixation cross (baseline). A high-pass filter of 256 s was applied. For the second-level analyses, task-related activations were first identified for the entire group using an ANOVA model with a main effect of task with 4 levels: *compare* > *baseline, rotate easy* > *baseline*, *rotate medium* > *baseline,* and *rotate hard* > *baseline*. On the group level, a voxel-wise height threshold of *p* < 0.001 (uncorrected) combined with a cluster-level extent threshold of *p* < 0.05 (corrected for multiple comparisons using the family wise error rate) was employed for whole-brain analyses.

Whole-brain group differences were assessed using an ANOVA model where the four groups were compared on the *rotate (easy* + *medium* + *hard)* > *baseline* contrast. Beta values for the clusters in which group differences were revealed by the F-test were then extracted using MarsBar (https://marsbar.sourceforge.net/index.html). A one-way ANOVA with Tukey’s Honest Significant Difference pairwise comparisons was performed using the R software^70^ to assess the character and directions of group differences in neural activations (beta values) in these clusters. The post-hoc analyses were corrected for multiple comparisons using Bonferroni correction. Behavioural results were visualised using the ggplot2 package for R^71^. Imaging results were visualised using MRIcroGL (https://www.nitrc.org/projects/mricrogl/).

The study was approved by the Ethics Board of the Faculty of Psychology, University of Warsaw. All procedures performed in this study were in accordance with the 1964 Helsinki declaration and its later amendments. All participants gave written informed consent to participate in the study.

## Supplementary information

Supplementary Information

## Data Availability

Data is available upon direct request to the corresponding author.
