# Supplementary material for: Male sexual orientation, gender nonconformity, and neural activity during mental rotations: an fMRI study
Source: Sci Rep. 2020 Oct 30;10:18709. doi: 10.1038/s41598-020-74886-0 (PMC7599322; doi:10.1038/s41598-020-74886-0)
Supplement: Supplementary file 1 — Supplementary Information [file 41598_2020_74886_MOESM1_ESM.docx]

**Supplementary Material**

**Male Sexual Orientation, Gender Nonconformity, and Neural Activity during Mental Rotations: An fMRI Study.**

**Monika Folkierska-Żukowska**^1*^**, Qazi Rahman**^2^**, Artur Marchewka**^3^**, Marek Wypych**^3^**, Dawid Droździel**^3^**, Andrzej Sokołowski**^4^**, Wojciech Ł. Dragan**^1^

^1^Faculty of Psychology, University of Warsaw, Warsaw, Poland

^2^Department of Psychology, Institute of Psychiatry, Psychology & Neuroscience, King’s College London, London, UK

^3^Laboratory of Brain Imaging, Nencki Institute of Experimental Biology of the Polish Academy of Sciences, Warsaw, Poland

^4^Department of Neurology, Memory and Aging Center, UCSF Weill Institute for Neurosciences, University of California, San Francisco, San Francisco, CA, USA

*Correspondence and requests for materials should be addressed to M.F.Z

(e-mail: [mfz@psych.uw.edu.pl)](mailto:wdragan@psych.uw.edu.pl))

**Behavioral Study**

**Methods**

*Participants* A total of 720 men who were either gay (*n* = 430, mean age: 28.22, *SD* = 6.80) or heterosexual (*n* = 290, mean age: 27.71, *SD* = 6.90) took part in the study.

*Recruitment procedures*

Participants were recruited in Poland via an online survey completed as a part of a larger project concerning correlates of sexual orientation. The survey was distributed through Facebook (paid adverts and organic traffic), leaflets, posters, and word of mouth. The survey was completed by 3,337 individuals between December 2016 and June 2019. Of these, 2,168 fulfilled the inclusion criteria (being a predominantly heterosexual or predominantly gay cis-gender man, aged 18–45 years) and were invited to the laboratory. Of these, 746 accepted the invitation and attended the laboratory (440 gay men and 306 heterosexual men). Fourteen individuals were excluded due to technical problems during the test and 12 due to reporting a sexual orientation different from that assessed in the on-line survey. The final sample consisted of 720 participants (430 gay and 290 heterosexual men). All participants who attended the laboratory received remuneration (approximately 8 EUR).

*Behavioural measures*

Sexual orientation and recalled childhood gender nonconformity were assessed in the same way as in the study described in the main text. See Table s1 for frequencies of answers on the Sell Assessment of Sexual Orientation in the two sexual orientation groups.

| **Table s1.** | |  |  |  |  |
| --- | --- | --- | --- | --- | --- |
| *Frequencies of answers on the Sell Assessment of Sexual Orientation in the two sexual orientation groups.* | | | | | |
|  |  | straight  (n = 290) | % | gay  (n = 430) | % |
| **I Sexual Attractions** | |  |  |  |  |
|  | ***During the past year, how many different men were you sexually attracted to:*** | | | | |
|  | 'none' | 276 | 95.17% | 0 | 0.00% |
|  | '1' | 14 | 4.83% | 6 | 1.40% |
|  | '2' | 0 | 0.00% | 7 | 1.63% |
|  | '3-5' | 0 | 0.00% | 60 | 13.95% |
|  | ' 6-10' | 0 | 0.00% | 77 | 17.91% |
|  | '11-49' | 0 | 0.00% | 141 | 32.79% |
|  | ' 50-99' | 0 | 0.00% | 75 | 17.44% |
|  | '100 or more' | 0 | 0.00% | 64 | 14.88% |
|  | ***During the past year, on average, how often were you sexually attracted to a man:*** | | | | |
|  | 'Never.' | 269 | 92.76% | 0 | 0.00% |
|  | 'Less than 1 time per month.' | 21 | 7.24% | 10 | 2.33% |
|  | '1-3 times per month' | 0 | 0.00% | 32 | 7.44% |
|  | '1 time per week.' | 0 | 0.00% | 35 | 8.14% |
|  | '2-3 times per week.' | 0 | 0.00% | 103 | 23.95% |
|  | '4-6 times per week.' | 0 | 0.00% | 94 | 21.86% |
|  | 'Daily.' | 0 | 0.00% | 156 | 36.28% |
|  | ***During the past year, the most I was sexually attracted to a man was:*** | | | | |
|  | 'Not at all sexually attracted.' | 254 | 87.59% | 0 | 0.00% |
|  | 'Slightly sexually attracted.' | 31 | 10.69% | 0 | 0.00% |
|  | 'Mildly sexually attracted.' | 5 | 1.72% | 0 | 0.00% |
|  | 'Moderately sexually attracted. ' | 0 | 0.00% | 15 | 3.49% |
|  | 'Significantly sexually attracted.' | 0 | 0.00% | 63 | 14.65% |
|  | 'Very sexually attracted.' | 0 | 0.00% | 135 | 31.40% |
|  | 'Extremely sexually attracted.' | 0 | 0.00% | 217 | 50.47% |
|  | ***During the past year, how many different women were you sexually attracted to?*** | | | | |
|  | 'none' | 0 | 0.00% | 397 | 92.33% |
|  | '1' | 17 | 5.86% | 33 | 7.67% |
|  | '2' | 12 | 4.14% | 0 | 0.00% |
|  | '3-5' | 52 | 17.93% | 0 | 0.00% |
|  | ' 6-10' | 55 | 18.97% | 0 | 0.00% |
|  | '11-49' | 73 | 25.17% | 0 | 0.00% |
|  | ' 50-99' | 31 | 10.69% | 0 | 0.00% |
|  | '100 or more' | 50 | 17.24% | 0 | 0.00% |
|  | ***During the past year, on average, how often were you sexually attracted to a woman?*** | | | | |
|  | 'Never.' | 0 | 0.00% | 392 | 91.16% |
|  | 'Less than 1 time per month.' | 9 | 3.10% | 37 | 8.60% |
|  | '1-3 times per month' | 27 | 9.31% | 0 | 0.00% |
|  | '1 time per week.' | 21 | 7.24% | 0 | 0.00% |
|  | '2-3 times per week.' | 64 | 22.07% | 0 | 0.00% |
|  | '4-6 times per week.' | 69 | 23.79% | 0 | 0.00% |
|  | 'Daily.' | 100 | 34.48% | 0 | 0.00% |
|  | ***During the past year, the most I was sexually attracted to a woman was:*** | | | | |
|  | 'Not at all sexually attracted.' | 0 | 0.00% | 376 | 87.44% |
|  | 'Slightly sexually attracted.' | 1 | 0.34% | 40 | 9.30% |
|  | 'Mildly sexually attracted.' | 4 | 1.38% | 14 | 3.26% |
|  | 'Moderately sexually attracted. ' | 19 | 6.55% | 0 | 0.00% |
|  | 'Significantly sexually attracted.' | 43 | 14.83% | 0 | 0.00% |
|  | 'Very sexually attracted.' | 67 | 23.10% | 0 | 0.00% |
|  | 'Extremely sexually attracted.' | 156 | 53.79% | 0 | 0.00% |
| **II Sexual Conduct** | |  |  |  |  |
|  | ***During the past year, how many different men did you have sexual contact with?*** | | | | |
|  | 'none' | 288 | 99.31% | 22 | 5.12% |
|  | '1' | 2 | 0.69% | 126 | 29.30% |
|  | '2' | 0 | 0.00% | 38 | 8.84% |
|  | '3-5' | 0 | 0.00% | 90 | 20.93% |
|  | ' 6-10' | 0 | 0.00% | 76 | 17.67% |
|  | '11-49' | 0 | 0.00% | 64 | 14.88% |
|  | ' 50-99' | 0 | 0.00% | 10 | 2.33% |
|  | '100 or more' | 0 | 0.00% | 4 | 0.93% |
|  | ***During the past year, on average, how often did you have sexual contact with a man?*** | | | | |
|  | 'Never.' | 290 | 100.00% | 22 | 5.12% |
|  | 'Less than 1 time per month.' | 0 | 0.00% | 86 | 20.00% |
|  | '1-3 times per month' | 0 | 0.00% | 131 | 30.47% |
|  | '1 time per week.' | 0 | 0.00% | 81 | 18.84% |
|  | '2-3 times per week.' | 0 | 0.00% | 86 | 20.00% |
|  | '4-6 times per week.' | 0 | 0.00% | 19 | 4.42% |
|  | 'Daily.' | 0 | 0.00% | 5 | 1.16% |
|  | ***During the past year, how many different women did you have sexual contact with?*** | | | | |
|  | 'none' | 38 | 13.10% | 424 | 98.60% |
|  | '1' | 175 | 60.34% | 6 | 1.40% |
|  | '2' | 26 | 8.97% | 0 | 0.00% |
|  | '3-5' | 35 | 12.07% | 0 | 0.00% |
|  | ' 6-10' | 8 | 2.76% | 0 | 0.00% |
|  | '11-49' | 6 | 2.07% | 0 | 0.00% |
|  | ' 50-99' | 0 | 0.00% | 0 | 0.00% |
|  | '100 or more' | 2 | 0.69% | 0 | 0.00% |
|  | ***During the past year, on average, how often did you have sexual contact with a woman?*** | | | | |
|  | 'Never.' | 37 | 12.76% | 424 | 98.60% |
|  | 'Less than 1 time per month.' | 42 | 14.48% | 6 | 1.40% |
|  | '1-3 times per month' | 77 | 26.55% | 0 | 0.00% |
|  | '1 time per week.' | 55 | 18.97% | 0 | 0.00% |
|  | '2-3 times per week.' | 54 | 18.62% | 0 | 0.00% |
|  | '4-6 times per week.' | 21 | 7.24% | 0 | 0.00% |
|  | 'Daily.' | 4 | 1.38% | 0 | 0.00% |
| **III Sexual Identity** | |  |  |  |  |
|  | ***I consider myself*** | | | | |
|  | 'Not at all homosexual.' | 229 | 78.97% | 0 | 0.00% |
|  | 'Slightly homosexual.' | 61 | 21.03% | 0 | 0.00% |
|  | Mildly homosexual.' | 0 | 0.00% | 0 | 0.00% |
|  | 'Moderately homosexual.' | 0 | 0.00% | 0 | 0.00% |
|  | 'Significantly homosexual.' | 0 | 0.00% | 0 | 0.00% |
|  | 'Very homosexual.' | 0 | 0.00% | 99 | 23.02% |
|  | 'Extremely homosexual.' | 0 | 0.00% | 331 | 76.98% |
|  | ***I consider myself*** | | | | |
|  | 'Not at all heterosexual.' | 0 | 0.00% | 343 | 79.77% |
|  | 'Slightly heterosexual.' | 0 | 0.00% | 86 | 20.00% |
|  | Mildly heterosexual.' | 0 | 0.00% | 0 | 0.00% |
|  | 'Moderately heterosexual.' | 0 | 0.00% | 0 | 0.00% |
|  | 'Significantly heterosexual.' | 0 | 0.00% | 0 | 0.00% |
|  | 'Very heterosexual.' | 82 | 28.28% | 0 | 0.00% |
|  | 'Extremely heterosexual.' | 208 | 71.72% | 0 | 0.00% |

For mental rotation, a computerised version (prepared for this study) of the Vandenberg and Kuse Mental Rotation Test was used^1^. This test is composed of 24 trials where the participant must identify which two of four figures are identical to the target figure (but rotated at an angle). Each trial was presented on a separate screen. Accuracy was the outcome variable. For each trial the participant received +1 point for selecting a correct figure and -1 point for selecting an incorrect figure; thus, the score for each trial ranged between -2 and 2. The tasks are preceded by instructions (translated into Polish), which include four unscored training tasks.

*Statistical methods*

To test the differences between gay and heterosexual men in age, years of education, and CGN, the normality of distribution was first assessed using the Kolmogorov-Smirnov test. As the variables were not normally distributed, the differences between groups were assessed using the Mann-Whitney test. A linear regression analysis was performed to test the hypothesis about the relationship between sexual orientation and CGN, and mental rotation performance. All variables have been standardised before entering into the model, and outliers were identified by calculating Mahalanobis distance, and applying the appropriate cut-off points. Mental Rotation score was the dependent variable, and age, years of education, CGN, sexual orientation, as well as an interaction between CGN and sexual orientations were entered as independent variables. All analyses were performed in IBM^Ⓡ^ SPSS^Ⓡ^ Statistics (Version 25).

**Results**

*Group characteristics*

Gay and heterosexual men did not differ in terms of age (*U* = 58802.50, *p* = .194), years of education (mean = 16.22, *SD* = 3.12, and mean = 16.63, *SD* = 3.15 respectively, *U* = 59206.50, *p* = .248), but did differ in terms of CGN scores as expected (mean = 3.63, *SD* = 0.49, and mean = 4.13, *SD* = 0.32, respectively; *U* = 23836.50, *p* < .001).

*The relationship between sexual orientation and CGN and mental rotation performance*

A total of 714 participants (426 gay men, 288 heterosexual men) were included in the regression analysis, since 4 gay men and 2 heterosexual men were identified as outliers. While the results of this analysis revealed that age accounts for some of the variance in performance, it also shows that CGN, but not sexual orientation, nor the interaction between the two explains some of this variance. The final regression model is presented in Table s2.

| **Table s2.** |  |  |  |  |  |
| --- | --- | --- | --- | --- | --- |
| *The relationship between sexual orientation and CGN and mental rotation performance—regression analysis* | | | | | |
|  | Unstandardized Coefficients | |  |  |  |
|  | B | Std. Error | Beta | *t* | *p*-value |
| Age (Years) | -0.11 | 0.04 | -0.11 | -2.61 | 0.009 |
| Education(Years) | 0.08 | 0.04 | 0.08 | 1.76 | 0.079 |
| CGN | 0.1 | 0.05 | 0.1 | 2 | 0.049 |
| Sexual Orientation | -0.05 | 0.1 | 0.02 | -0.46 | 0.635 |
| CGN * Sexual Orientation | -0.04 | 0.1 | -0.02 | -0.35 | 0.729 |

**fMRI Study**

*Group characteristics*

Group differences in terms of age, IQ scores and CGN scores are presented in the results section in the main text. Table s3 presents means and standard deviations for age, IQ scores, and CGN scores for the four groups, corresponding to what is graphically represented in Figure 1 in the main text.

| **Table s3.** |  |  |  |  |  |  |
| --- | --- | --- | --- | --- | --- | --- |
| *Means and standard deviations for Age, IQ scores, and CGN scores for the four groups* | | | | | |  |
|  | Age | | IQ | | CGN | |
|  | mean | SD | mean | SD | mean | SD |
| Childhood gender conforming heterosexual women (*n* = 22) | 26.24 | 3.53 | 46.95 | 4.34 | 3.1 | 0.26 |
| childhood gender non-conforming gay men (*n* = 23) | 26.96 | 4.04 | 47.26 | 4.14 | 2.93 | 0.26 |
| Childhood gender conforming gay men (*n* = 23) | 26.26 | 4.48 | 46.61 | 3.84 | 4.08 | 0.2 |
| Childhood gender conforming heterosexual men (*n* = 22) | 25.59 | 3.65 | 45.68 | 6.18 | 4.03 | 0.16 |

*Behavioural Results*

Group differences in terms of performance in mental rotation in the scanner are presented in the results section in the main text. Table s4 presents means and standard deviations for scores and reaction times in each task condition for the four groups, corresponding to what is graphically represented in Figure 2 in the main text.

| **Table s4.** | |  |  |  |  |  |  |  |  |
| --- | --- | --- | --- | --- | --- | --- | --- | --- | --- |
| *Means and standard deviations for scores and reaction times for the mental rotation task performed in the scanner for each group at each difficulty level* | | | | | | | | | |
|  |  | Childhood gender conforming heterosexual women | | Childhood gender non-conforming gay men | | Childhood gender conforming gay men | | Childhood gender conforming heterosexual men. | |
|  |  | mean | SD | mean | SD | mean | SD | mean | SD |
| Scores | | | | | | | | | |
|  | compare | 0.98 | 0.05 | 0.99 | 0.02 | 0.98 | 0.02 | 0.96 | 0.08 |
|  | easy | 0.92 | 0.05 | 0.92 | 0.08 | 0.92 | 0.08 | 0.93 | 0.07 |
|  | medium | 0.79 | 0.07 | 0.81 | 0.1 | 0.79 | 0.11 | 0.8 | 0.16 |
|  | hard | 0.71 | 0.1 | 0.71 | 0.13 | 0.72 | 0.14 | 0.7 | 0.14 |
| Reaction times (s) | | | | | | | | | |
|  | compare | 1.91 | 0.32 | 1.98 | 0.42 | 1.92 | 0.4 | 1.97 | 0.54 |
|  | easy | 2.59 | 0.36 | 2.71 | 0.39 | 2.63 | 0.42 | 2.51 | 0.51 |
|  | medium | 3.15 | 0.3 | 3.22 | 0.36 | 3.13 | 0.37 | 3.06 | 0.39 |
|  | hard | 3.22 | 0.27 | 3.36 | 0.37 | 3.28 | 0.34 | 3.31 | 0.35 |

Group differences in terms of strengths of activations in regions of interest during the performance of mental rotation in the scanner are presented in the results section in the main text. Table s5 presents the results of the region of interest analysis—comparisons between group mean beta values for a given structure in the four groups (*F*-tests and Tukey HSD comparisons with Bonferroni correction), corresponding to what is graphically represented in Figure 6 in the main text.

| **Table s5.** |  |  |  |  |  |  |  |  |  |
| --- | --- | --- | --- | --- | --- | --- | --- | --- | --- |
| *Region of interest analysis group differences in mean activations for all levels of difficulty  (rotation< baseline contrast) in the selected structures* | | | | | | | | | |
|  | Structure | L/R Precuneus | R Angular Gyrus | R Middle Temporal Gyrus | L/R Precentral /Paracentral Gyrus | L Middle Temporal Gyrus | R Amygdala /Parahippocampal Gyrus | L Medial Superior Frontal Gyrus | R Superior Frontal Gyrus |
|  | F-test | F(3,86) = 13.28 | F(3,86) = 12.38 | F(3,86)=9.17 | F(3,86)=9.96 | F(3,86)=10.51 | F(3,86)=10.72 | F(3,86)=10.86 | F(3,86)=12.23 |
|  |  | p>0.001 | p>0.001 | p>0.001 | p>0.001 | p>0.001 | p>0.001 | p>0.001 | p>0.001 |
| Comparison (Tukey HSD) |  |  |  |  |  |  |  |  |  |
| Childhood gender conforming heterosexual men - Childhood gender conforming heterosexual women | difference | 2.43 | 2.11 | 1.41 | 1.61 | 1.49 | 1.34 | 1.22 | 1.28 |
|  | p adjusted | >0.001 | 0.008 | >0.001 | >0.001 | >0.001 | 0.022 | 0.001 | 0.000 |
| Gender nonconforming gay men - Childhood gender conforming heterosexual women | difference | 1.32 | 0.17 | 0.29 | 1.54 | 0.46 | 0.09 | 0.98 | 0.82 |
|  | p adjusted | 0.197 | 1.000 | 1.000 | 0.001 | 1.000 | 0.600 | 0.019 | 1.000 |
| Childhood gender conforming gay men - Childhood gender conforming heterosexual women | difference | 2.53 | 2.56 | 1.08 | 1.33 | 1.19 | 2.01 | 1.41 | 1.53 |
|  | p adjusted | 0.000 | 0.000 | 0.021 | 0.007 | 0.005 | >0.001 | >0.001 | >0.001 |
| Childhood gender conforming heterosexual men - Gender nonconforming gay men | difference | 1.11 | 1.93 | 1.11 | 0.08 | 1.03 | 0.44 | 0.24 | 0.46 |
|  | p adjusted | 0.623 | 0.020 | 0.014 | 1.000 | 0.032 | 1.000 | 1.000 | 1.000 |
| Childhood gender conforming gay men - Childhood gender conforming heterosexual men. | difference | -0.10 | -0.46 | 0.33 | 0.28 | 0.30 | -0.68 | -0.19 | -0.25 |
|  | p adjusted | 1.000 | 1.000 | 1.000 | 1.000 | 1.000 | 1.000 | 1.000 | 1.000 |
| Childhood gender conforming gay men - Gender nonconforming gay men | difference | 1.21 | 2.39 | 0.79 | -0.20 | 0.74 | 1.12 | 0.43 | 0.71 |
|  | p adjusted | 0.338 | 0.001 | 0.344 | 1.000 | 0.497 | 0.105 | 1.000 | 0.320 |

**References**

1. [Vandenberg, S. G. & Kuse, A. R. Mental rotations, a group test of three-dimensional spatial visualization. *Percept. Mot. Skills* **47**, 599–604 (1978).](http://paperpile.com/b/jz3Ewl/WOOH)
